# Comparative Analysis of Metagenomics and Metataxonomics for the Characterization of Vermicompost Microbiomes

**DOI:** 10.3389/fmicb.2022.854423

**Published:** 2022-05-10

**Authors:** Marcos Pérez-Losada, Dhatri Badri Narayanan, Allison R. Kolbe, Ignacio Ramos-Tapia, Eduardo Castro-Nallar, Keith A. Crandall, Jorge Domínguez

**Affiliations:** ^1^Computational Biology Institute, The George Washington University, Washington, DC, United States; ^2^Department of Biostatistics and Bioinformatics, Milken Institute School of Public Health, The George Washington University, Washington, DC, United States; ^3^CIBIO-InBIO, Centro de Investigação em Biodiversidade e Recursos Genéticos, Universidade do Porto, Vairão, Portugal; ^4^Instituto de Investigación Interdisciplinaria (I3), Universidad de Talca, Talca, Chile; ^5^Departamento de Microbiología, Facultad de Ciencias de la Salud, Universidad de Talca, Talca, Chile; ^6^Grupo de Ecoloxía Animal (GEA), Universidade de Vigo, Vigo, Spain

**Keywords:** earthworm, grape marc, ITS, metagenomics, metataxonomics, microbiome, vermicompost, 16S rRNA

## Abstract

The study of microbial communities or microbiotas in animals and environments is important because of their impact in a broad range of industrial applications, diseases and ecological roles. High throughput sequencing (HTS) is the best strategy to characterize microbial composition and function. Microbial profiles can be obtained either by shotgun sequencing of genomes, or through amplicon sequencing of target genes (e.g., 16S rRNA for bacteria and ITS for fungi). Here, we compared both HTS approaches at assessing taxonomic and functional diversity of bacterial and fungal communities during vermicomposting of white grape marc. We applied specific HTS workflows to the same 12 microcosms, with and without earthworms, sampled at two distinct phases of the vermicomposting process occurring at 21 and 63 days. Metataxonomic profiles were inferred in DADA2, with bacterial metabolic pathways predicted *via* PICRUSt2. Metagenomic taxonomic profiles were inferred in PathoScope, while bacterial functional profiles were inferred in Humann2. Microbial profiles inferred by metagenomics and metataxonomics showed similarities and differences in composition, structure, and metabolic function at different taxonomic levels. Microbial composition and abundance estimated by both HTS approaches agreed reasonably well at the phylum level, but larger discrepancies were observed at lower taxonomic ranks. Shotgun HTS identified ~1.8 times more bacterial genera than 16S rRNA HTS, while ITS HTS identified two times more fungal genera than shotgun HTS. This is mainly a consequence of the difference in resolution and reference richness between amplicon and genome sequencing approaches and databases, respectively. Our study also revealed great differences and even opposite trends in alpha- and beta-diversity between amplicon and shotgun HTS. Interestingly, amplicon PICRUSt2-imputed functional repertoires overlapped ~50% with shotgun Humann2 profiles. Finally, both approaches indicated that although bacteria and fungi are the main drivers of biochemical decomposition, earthworms also play a key role in plant vermicomposting. In summary, our study highlights the strengths and weaknesses of metagenomics and metataxonomics and provides new insights on the vermicomposting of white grape marc. Since both approaches may target different biological aspects of the communities, combining them will provide a better understanding of the microbiotas under study.

## Introduction

High throughput sequencing (HTS) has revolutionized our ability to survey microbial communities through DNA sequencing. Currently, there are two main HTS strategies for the analysis of microbial communities on Next-Generation Sequencing (NGS) platforms: targeted amplicon sequencing and shotgun metagenomics. Targeted amplicon sequencing (e.g., Bybee et al., 2011) or metataxonomics (as defined in Marchesi and Ravel, 2015) allows for efficiently running large volumes of samples at relatively low costs compared to traditional Sanger sequencing of PCR products. Specific gene regions have been effectively developed to survey various members of the microbial community. The 16S ribosomal RNA (rRNA) gene has been effectively used to characterize bacterial diversity (Klindworth et al., 2013; Bowman and Ducklow, 2015) and predict its metabolic function by imputation (Langille et al., 2013), in no small part due to extensive reference databases that have been collected over the past 25 years (e.g., Quast et al., 2013; Cole et al., 2014). Similarly, the internal transcribed spacer (ITS) region of the rRNA operon for fungi (Nilsson et al., 2019a) is an effective DNA barcode (Schoch et al., 2012) to characterize fungal diversity also due to an extensive database of reference sequences (Merget et al., 2012; Santamaria et al., 2018; Nilsson et al., 2019b). However, despite their widespread use in microbial diversity studies, these two target genes are not as effective at classifying sequences at the species and strain levels (Vetrovsky et al., 2016; Johnson et al., 2019; Strube, 2021).

Shotgun metagenomics is an alternative HTS approach that does not rely on the PCR amplification of a single gene of the bacterial or fungal genome, but in the direct sequencing of DNA isolated from the whole microbial community or microbiota (prokaryotes, eukaryotes and viruses). This approach generates small DNA fragments or reads which are then sorted and classified bioinformatically (e.g., Hong et al., 2014; Wood et al., 2019) by mapping reads to reference genomes (reviewed in Miossec et al., 2020). This metagenomic approach has the advantages of broader taxonomic range and microbial diversity capacity, greater resolution [often species and even strains (e.g., Francis et al., 2013)], and greater potential for functional insights due to the mapping of sequenced reads across the entire genome. The drawbacks of the metagenomic approach are that it is highly dependent on the reference database used (Nasko et al., 2018; Breitwieser et al., 2019)—unless computationally intense metagenomic assembly-based approaches are used, the cost per sample is substantially higher than for metataxonomics, and requires sophisticated computational methods and powerful hardware resources for their analysis (Gevers et al., 2012; Miossec et al., 2017). Nevertheless, both metataxononomic and metagenomic approaches perform well compared to traditional methods of microbial culture identification (Hilton et al., 2016) and allow for a robust characterization of microbiome diversity (Miossec et al., 2020).

Given that both metataxonomics and metagenomics provide useful and often complementary data and insights, we applied both approaches to characterize the diversity of the bacterial and fungal communities associated with vermicomposting. Vermicomposting takes advantage of the synergistic effects of worms and microorganisms (bacteria and fungi) to decompose organic waste (Ali et al., 2015). At the same time, worms can enrich the microbial communities of the resulting compost, enhancing subsequent production as a richer form of organic fertilizer, thus offering a sustainable alternative to chemicals while converting waste products into fertilizer. Our study specifically focuses on vermicomposting in viticulture. Vermicomposting of grape marc (the solid portion of the grape must) can effectively produce a high-quality organic fertilizer that is nutrient-rich, microbially diverse, and a source of bioactive polyphenols (Dominguez et al., 2014). It also reduces environmentally hazardous waste (due to low pH and high polyphenol content) from the wine making process. Our previous work on this system used white grape marc (*Vitis vinifera* L. cv. Albariño) from wineries in the northwest part of Spain (Galicia). Using a 16S rRNA metataxonomic approach, we showed increasing bacterial diversity (alpha and beta) over time (out to 91 days with significant changes after 7 days) (Kolbe et al., 2019).

This new study uses our white grape marc system (although, from a different vineyard) to test for changes in diversity over time (21 and 63 days) with and without (control) earthworms. We use metataxonomic approaches to characterize both bacterial and fungal diversity as well as shotgun metagenomic sequencing for broad characterization of the vermicomposting microbiome. These different sequencing strategies provide us with a detailed understanding of changes in microbial communities over time during vermicomposting compared to composting (no earthworms). Moreover, HTS of vermicompost has mainly focused on 16S rRNA metataxonomics; but now that ITS (e.g., Dominguez et al., 2021) and shotgun (Huang et al., 2020) sequencing are starting to be used to characterize the vermicompost microbiota, comparing the outcomes of the specific HTS strategies used here will guide further research on this topic.

## Materials and Methods

### Vermicomposting Microcosm Design and Sampling

White grape marc (*Vitis vinifera* L. cv. Albariño) was provided by Terras Gauda, a winery located in southern Galicia (northwestern Spain). Grape marc was stored at 4°C until needed and turned and moistened with water for 2 days prior to the vermicomposting trial. Microcosm reactors were constructed with PVC as cylinders with a 30 cm diameter and 70 cm height. Each microcosm was initiated with a base layer of vermicompost from grape marc that acted as a bed for the earthworms. Three microcosms were inoculated with 400 earthworms (EW) (*Eisenia andrei*) and three were left without earthworms (control—CT). Fresh grape marc (2 kg fresh weight) was added weekly with a mesh (5 mm pore size) between weekly grape marc additions. The mesh allowed the earthworms to move freely throughout the cylinder but prevented fresh material from being mixed with earthworm-processed material. All of the reactors were kept in an air-conditioned room with temperature set to 20°C throughout the duration of the trial.

Vermicomposting involves an active phase, where earthworm activity is critical, and a maturation phase, which takes place once worms leave the substrate, where microorganisms are the impactful community members. The active phase comprises all the processes associated with the passage of substrate through the earthworm gut (GAPs: gut-associated processes). During this phase, earthworm digestion reduces microbial biomass and activity and modifies the structure and function of the microbial communities. In the maturation phase, earthworm excreted materials or casts start aging, while their associated microbial communities experience a turnover (i.e., cast-associated processes, CAPs). Our microcosms resembled a time profile consisting of layers of increasing age where time 21 days represents the active phase of vermicomposting (GAP) and time 63 days represents the maturation phase (CAP). After 63 days microcosms were dismantled. From each layer, earthworms were removed manually from the substrate. We sampled two microcosm substrate layers representing the active (time 21 days) and mature (time 63 days) phases of vermicomposting. We collected 3 samples from each layer at random and mixed them gently prior to analysis. Our balanced experimental design thus included a total of 12 samples distributed across four groups with three biological replicates per group. Those four groups corresponded to two treatments, with (EW) and without earthworms (control; CT), and two times or ages (21 and 63 days).

### DNA Extraction and Sequencing

DNA was extracted from 0.25 g of distilled grape marc using the MO-BIO PowerSoil® kit (MoBio Laboratories Inc., Carlsbad, California) according to manufacturer's protocols. DNA quality and quantity were determined using BioTek's Take3TM Multi-Volume Plate (SinergyTM 2 Multi-Mode Microplate Reader, Bio-Tek Instruments, Inc.), as previously described in Kolbe et al. (2019). Aliquots of the same 12 DNA extractions were sequenced using standard shotgun and amplicon (16S rRNA and ITS) HTS strategies. Shotgun DNA libraries were prepared using the KAPA Hyper Prep Kit (Kapa Biosystems, Wilmington, MA), as per the manufacturer's instructions. Library quality was validated using the Agilent 2100 Bioanalyzer system and subjected to two runs of paired-end sequencing (2 × 125 bp) on an Illumina HiSeq2500 platform at the University of Southern California genomics core. All genomic libraries were sequenced twice and reads merged for downstream analysis.

Amplicons were amplified and sequenced following the protocols used in the Earth Microbiome Project (Thompson et al., 2017)—https://earthmicrobiome.org. The bacterial microbiota (bacteriota) was characterized using the 16S rRNA V4 region (~250 bp)—primer 515F (Parada) Fwd: GTGYCAGCMGCCGCGGTAA and primer 806R (Apprill) Rev: GGACTACNVGGGTWTCTAAT; while the fungal microbiota (mycobiota) was characterized using a ~150 bp region of the ITS gene (ITS1)—primer ITS1F Fwd: CTTGGTCATTTAGAGGAAGTAA and primer ITS2 Rev: GCTGCGTTCTTCATCGATGC. PCR and library amplicons were validated using a Thermo Fisher Qubit 3.0 fluorometer High-Sensitivity DNA kit and an Agilent Bioanalyzer High-Sensitivity DNA kit. QC amplicons were sequenced in an Illumina MiSeq platform (reagent kit v2−500-cycles) at the Argonne National Laboratory IL using a dual-index sequencing strategy (Kozich et al., 2013). These platform and protocols are routinely applied in HTS.

### Metataxonomic Analysis

We applied robust and validated bioinformatic pipelines commonly used in the analysis of amplicon data. DNA amplicons were processed using the R package dada2 (Callahan et al., 2016) to infer amplicon sequence variants (ASVs) present in each sample. ASVs provide a more accurate, consistent, and reproducible description of amplicon-sequenced communities than is possible with operational taxonomic units (OTUs) defined at a constant level (97% or other) of sequence similarity (Callahan et al., 2016, 2017). 16S rRNA raw reads were filtered using standard parameters with no uncalled bases, a maximum of 2 expected errors, and truncating reads at a quality score of 2 or less. Taxonomic assignment for 16S rRNA was performed against the Silva v132 database (Quast et al., 2013) using the dada2-formatted training files for taxonomy and up to the genus-level assignment (Callahan et al., 2016). ITS raw reads were first subjected to adapter trimming by cutadapt (Martin, 2011), which removed primer sequences due to read-through. Filtering was performed as described above, with the additional parameter of minimum read length of 50 bp. Taxonomic assignment for ITS was performed against the UNITE v18.11.2018 database (Nilsson et al., 2019b). ASVs were aligned using MAFFT (Katoh and Standley, 2013) and used to estimate phylogenetic relationships with FastTree (Price et al., 2010; Piñeiro et al., 2020). The resulting data were imported into phyloseq (McMurdie and Holmes, 2013) for further analysis.

Bacterial metabolic pathways were predicted using 16S rRNA ASVs in PICRUSt2 (Douglas et al., 2020) following the standard pipeline. No such analysis is available for ITS amplicons, which precluded us from comparing metagenomic and metataxonomic approaches at estimating fungal metabolic profiles. The alignment software HHMER (Finn et al., 2011) was used to align reference sequences, which were then placed into a reference tree using EPA-NG (Barbera et al., 2019) and GAPPA (Czech et al., 2019). Hidden-state prediction was performed using castor (Louca and Doebeli, 2018). MetaCyc pathway level predictions were made with MinPath (Ye and Doak, 2009).

### Metagenomic Analysis

Shotgun HiSeq paired-end reads were processed in PRINSEQ-lite (Schmieder and Edwards, 2011) by trimming reads and bases with a <25 PHRED score and removing exact duplicates, reads with undetermined bases, and low complexity reads (dust filter = 30). After trimming, at least 93% of the reads remained in all samples. Taxonomic assignment was performed using PathoScope (Hong et al., 2014). Trimmed reads were aligned against bacterial and fungal RefSeq genomes using the PathoScope MAP module, which implements the bowtie2 read-mapping strategy (Langmead and Salzberg, 2012). Reads that aligned to the human (hg38) genome, phiX174, viruses, non-bacterial prokaryotes, non-fungal eukaryotes and plastid/mitochondrial RefSeq genomes were filtered from the final datasets. Taxonomic assignment was performed using the PathoScope ID module (Francis et al., 2013). The resulting datasets were imported into phyloseq (McMurdie and Holmes, 2013) for downstream analyses. PathoScope is a robust and validated method of metagenomic profiling of unassembled sequencing data (Miossec et al., 2017, 2020).

Bacterial metabolic functions were inferred using Humann2 (Franzosa et al., 2018). This is a well-known pipeline for efficiently and accurately profiling the abundance of microbial pathways in a community from metagenomic data. Moreover, because both Humann2 and PICRUSt2 use the Kyoto Encyclopedia of Genes and Genomes (KEGG) orthology (KO) accession numbers (Kanehisa et al., 2014), inferred (Humann2) and predicted (PICRUSt2) functional profiles can be directly compared. It is important, however, to keep in mind that these two approaches and databases were originally designed to characterize human microbiomes and related functions.

### Microbial Diversity and Statistical Analysis

We normalized all our samples using the negative binomial distribution as recommended by McMurdie and Holmes (2013) and implemented in the Bioconductor package DESeq2 (Love et al., 2014). This approach simultaneously accounts for library size differences and biological variability.

We compared taxonomic profiles of phyla, class, family and genus between shotgun metagenomics and 16S rRNA and ITS amplicon sequencing. It has been reported that for ~42% of bacterial genera there will be pairs of congeneric sequences that cannot be easily separated because of the high similarity of their 16S rRNA sequences (Vetrovsky and Baldrian, 2013; Strube, 2021). Hence, since species and strains are particularly challenging to identify (or unattainable) for the metataxonomic methods used here (Vetrovsky and Baldrian, 2013), and are not commonly reported in vermicomposting (e.g., Gopal et al., 2017; Dominguez et al., 2019), we did not consider lower-than-genus taxonomic levels in our analyses.

We compared metagenomic bacterial and fungal abundances and diversity indices to metataxonomic 16S rRNA bacterial and ITS fungal abundances, respectively. Taxonomic alpha-diversity was estimated using Chao1 richness and Shannon, Simpson and Fisher indices, which account for both richness and evenness. Beta-diversity was estimated using Bray-Curtis and Jaccard distances; these two non-phylogenetic indices are robust to sparse data in count tables, as often is the case for microbiome data. Taxonomic dissimilarity among samples was explored using principal coordinates analysis (PCoA), while functional differences were explored using principal component analysis (PCA).

We used linear regression to investigate associations between alpha-diversity indices and treatment (with and without earthworms) and age (21 and 63 days). We also tested for variation in phylum abundances across experimental groups; we chose this taxonomic category because of its larger concordance between HTS strategies (see Section Results). Significant differences in beta-diversity across treatments and ages were determined using the permutational multivariate analysis of variance (adonis function) implemented in the R package vegan.

The bioinformatic methods and parameters chosen represent an example of the methodologies and parameter settings commonly used in 16S rRNA and ITS metataxononic and metagenomic studies, including those of vermicompost microbiomes. Our results and insights are then closely connected to the specific analytical and molecular (see above) workflows used here. Other amplicon and shotgun protocols or analytical pipelines may lead to different results. Several methodological comparisons of shotgun and amplicon target sequencing approaches and analytical pipelines have been already published using different NGS platforms including real (natural and mock samples) and simulated sequences (McIntyre et al., 2017; Sczyrba et al., 2017; Nasko et al., 2018; Heeger et al., 2019; Ye et al., 2019; Zielezinski et al., 2019; Marcelino et al., 2020; Miossec et al., 2020). We refer the reader to previous literature on those topics for a thorough comparison of metataxononic and metagenomic HTS strategies.

## Results

Targeted amplicon sequencing resulted in 9,444 to 14,516 16S rRNA paired-end reads and 16,324 to 40,850 ITS paired-end reads per sample (Supplementary Table 1). After quality control, the 16S rRNA amplicon sequence datasets ranged from 7,638 to 12,206 16S rRNA sequences per sample, corresponding to 1,204 bacterial ASVs; while the ITS amplicon sequence datasets ranged from 7,543 to 28,817 ITS sequences per sample, corresponding to 196 fungal ASVs. For shotgun sequencing, raw paired-end reads ranged from 27,720,512 to 110,656,570 reads per sample, of which 1,638,115 to 6,147,003 reads per sample mapped to 943 bacterial genomes, and 13,263 to 170,019 reads per sample mapped to 33 fungal genomes (Supplementary Table 1). Rarefaction analyses of bacterial and fungal amplicon and shotgun data suggested adequate sample sizes (i.e., plateaued curves) for all the samples (Supplementary Figure 1).

### Taxonomic and Functional Diversity of Bacteriomes

**Figure 1:**
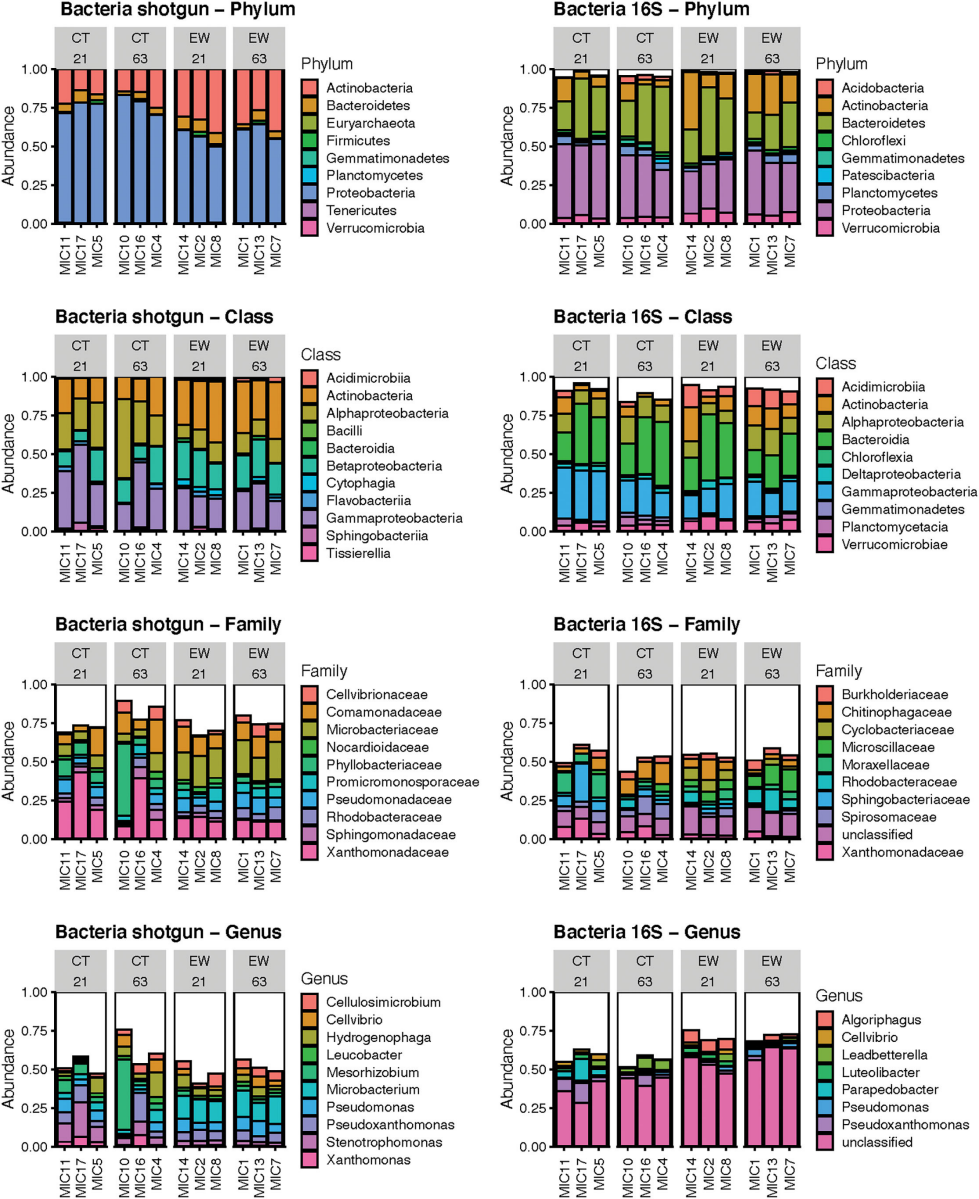
Bar plots of the mean relative abundance of the predominant bacteria by phylum, class, family and genus using shotgun reads and 16S ASVs in 12 microcosms (MIC). The white section of the bar plots represents the less abundant taxa aggregated.

The metagenomic approach detected 10 phyla, 22 classes, 143 families and 412 bacterial genera; while the 16S rRNA amplicon approach detected 24 phyla, 43 classes, 162 families and 235 bacterial genera. We excluded Archaea from further analyses as the proportion of Archaea 16S rRNA reads per sample varied between 0 and 2%. We compared mean relative microbial abundances between both HTS sequencing approaches to establish rankings for each taxonomic category (Figure 1; Supplementary Tables 2, 3). Both HTS approaches depicted Proteobacteria as the most abundant phylum followed by Actinobacteria or Bacteroidetes. The metagenomic approach put Firmicutes in the 4th ranking, while the metataxonomic approach put it in the 12th place, albeit with low abundance in both cases. Additional predominant phyla found by both analyses were unique to one methodology or varied in their rankings. Bacteroidia, Gammaproteobacteria, Alphaproteobacteria, Actinobacteria and Betaproteobacteria were among the four more abundant classes for at least one of the two HTS methods, but their rankings varied among them. At the family level, Xanthomonadaceae, Microbacteriaceae, Comamonadaceae and Phyllobacteriaceae dominated the metagenomic bacterial reads, while only Xanthomonadaceae was predominant in the metataxonomic approach, where the 1st family ranking was comprised of unclassified ASVs. A similar pattern was observed at the genus level, where *Microbacterium, Mesorhizobium, Pseudoxanthomonas* and *Stenotrophomonas* dominated the top-four metagenomic rankings, while only *Pseudoxanthomonas* was predominant among the 16S rRNA bacterial genera. As before, the 1st genus-ranking was composed of unclassified ASVs, which highlights the higher taxonomic resolution of the metagenomic approach over the metataxonomic approach.

We used linear regression to statistically compare the mean relative abundance of the four predominant bacterial phyla across treatments (with and without earthworms) and age (21 and 63 days) groups (Table 1). Eight out of the 12 linear model tests were non-significant in both HTS approaches. Proteobacteria and Actinobacteria varied significantly between treatments in both HTS approaches, while Proteobacteria varied significantly for the treatment and age interaction in the metataxonomic approach, and Bacteroidetes varied significantly between ages for the metagenomic approach.

**Table 1:** Linear model (LM) analyses of phylum abundances for bacteria and fungi from metatatoxonomic (16S and ITS) and metagenomic (MG) strategies.

	**16S**	**MG**	**ITS**
**Bacteria**			
Firmicutes			
Treatment	0.56	0.24	–
Age	0.93	0.64	–
Treatment^*^Age	0.51	0.00	–
Proteobacteria			
Treatment	13.61^**^	38.92^***^	–
Age	0.74	1.33	–
Treatment^*^Age	10.08^*^	0.18	–
Actinobacteria			
Treatment	8.68^*^	24.67^**^	–
Age	0.00	0.00	–
Treatment^*^Age	0.27	0.05	–
Bacteroidetes			
Treatment	0.33	3.14	–
Age	0.27	7.80^*^	–
Treatment^*^Age	2.19	0.24	–
**Fungi**			
Ascomycota			
Treatment	–	0.76	0.40
Age	–	0.98	1.30
Treatment^*^Age	–	1.01	1.38
Basidiomycota			
Treatment	–	0.95	0.32
Age	–	1.01	1.17
Treatment^*^Age	–	0.99	1.06

*The significance of LMs was estimated using ANOVA of Treatment III with Satterthwaite approximation for 1 degree of freedom. For each test, we report the relevant F statistic (F) and significance [P(>F)] as*

* *p < 0.05,*

** *p < 0.01,*

*** *p < 0.001. MG, metagenomic analysis*.

**Figure 2:**
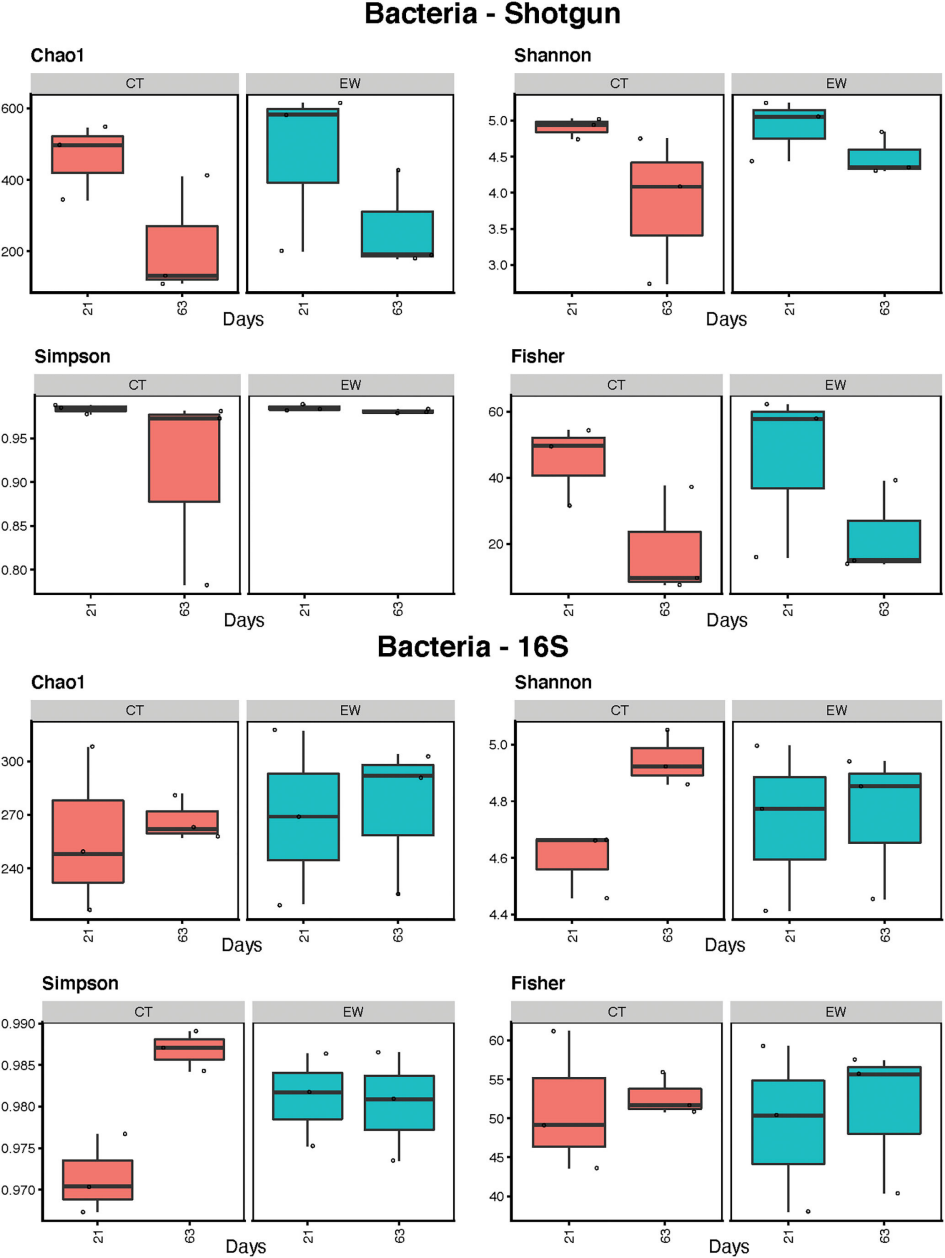
Bacterial alpha-diversity estimates (Chao1, Shannon, Simpson, and Fisher indices) for treatment (control, CT; earthworm, EW) and age (21 and 63 days) groups in shotgun metagenomic and 16S metataxonomic strategies.

Bacterial alpha-diversity of control (CT) and earthworm (EW) treatment samples was measured after 21 and 63 days using diversity indices of Chao1, Shannon, Simpson, and Fisher (Figure 2; Table 2). Diversity estimates ranges overlapped between metagenomic and metataxonomic data for the Shannon and Simpson indices, but larger differences were observed for Chao1 (higher average estimates for shotgun) and Fisher (lower average estimates for shotgun). Diversity trends, however, varied across all indices and most groups, with some even showing opposite trends (e.g., Shannon CT). Overall metagenomic alpha-diversity decreased over time for both CT and EW groups, while 16S rRNA alpha-diversity did not vary or increased overtime for the same two groups. Some of these trend differences were significant in our linear regression analyses (Table 2), but never for the same groups in both HTS strategies.

**Table 2:** Linear model (LM) analyses of alpha-diversity indices and permutational multivariate analysis of variance of beta-diversity indices from metatatoxonomic (16S and ITS) and metagenomic (MG) strategies (b, bacteria; f, fungi).

	**16S**	**MGb**	**ITS**	**MGf**
**Alpha-diversity**				
Chao1	267	353	51	11
Treatment	0.15	0.08	21.84^**^	0.08
Age	0.01	5.24	1.87	1.37
Treatment^*^Age	0.01	0.05	1.11	0.00
Age CT	0.12	4.56^*^	2.58	0.74
Age EW	0.02	1.62	0.05	0.63
Shannon	4.8	4.5	2.3	0.7
Treatment	0.07	0.96	26.45^***^	0.04
Age	2.35	4.76	2.78	0.88
Treatment^*^Age	1.83	0.90	−1.55	2.38
Age CT	15.63^*^	3.06	0.01	7.77^*^
Age EW	0.01	1.93	1.31	0.11
Simpson	0.98	0.97	0.80	0.32
Treatment	0.29	1.16	30.11^***^	0.50
Age	6.16^*^	1.35	0.41	0.74
Treatment^*^Age	7.62^*^	1.08	2.40	3.86
Age CT	24.66^**^	1.21	1.61	14.70^*^
Age EW	0.03	2.43	0.83	0.35
Fisher	51.1	32.9	6.7	1.1
Treatment	0.14	0.05	15.58^**^	0.13
Age	0.12	5.76^*^	3.13	1.42
Treatment^*^Age	0.003	0.04	0.96	0.02
Age CT	0.07	4.91^*^	3.22	0.84
Age EW	0.06	1.81	0.22	0.68
**Beta-diversity**				
Bray-Curtis				
Treatment	10.29^***^	3.62^*^	5.30^***^	0.25
Age	3.88^**^	3.39^*^	4.30^**^	1.16
Treatment^*^Age	2.89^*^	1.33	2.32	0.78
Age CT	4.06	2.79	6.92	0.83
Age EW	2.15	1.12	1.30	0.69
Jaccard				
Treatment	5.42^***^	2.89^**^	3.43^**^	0.47
Age	2.47^**^	2.33^*^	2.96^**^	1.52
Treatment^*^Age	2.13^*^	1.33	1.66	0.75
Age CT	2.60	2.09	4.26	0.82
Age EW	1.76	1.17	1.10	0.69

We compared treatment, age and their interaction (treatment*age) and ages within control (Age CT) and earthworm (Age EW) groups. The significance of LMs was estimated using ANOVA of Treatment III with Satterthwaite approximation for 1 degree of freedom. For each test, we report the relevant F statistic (F) and significance [P(>F)] as

* *p < 0.05,*

** *p < 0.01,*

*** *p < 0.001. Mean alpha-diversity estimates across all samples are also indicated in cursive*.

**Figure 3:**
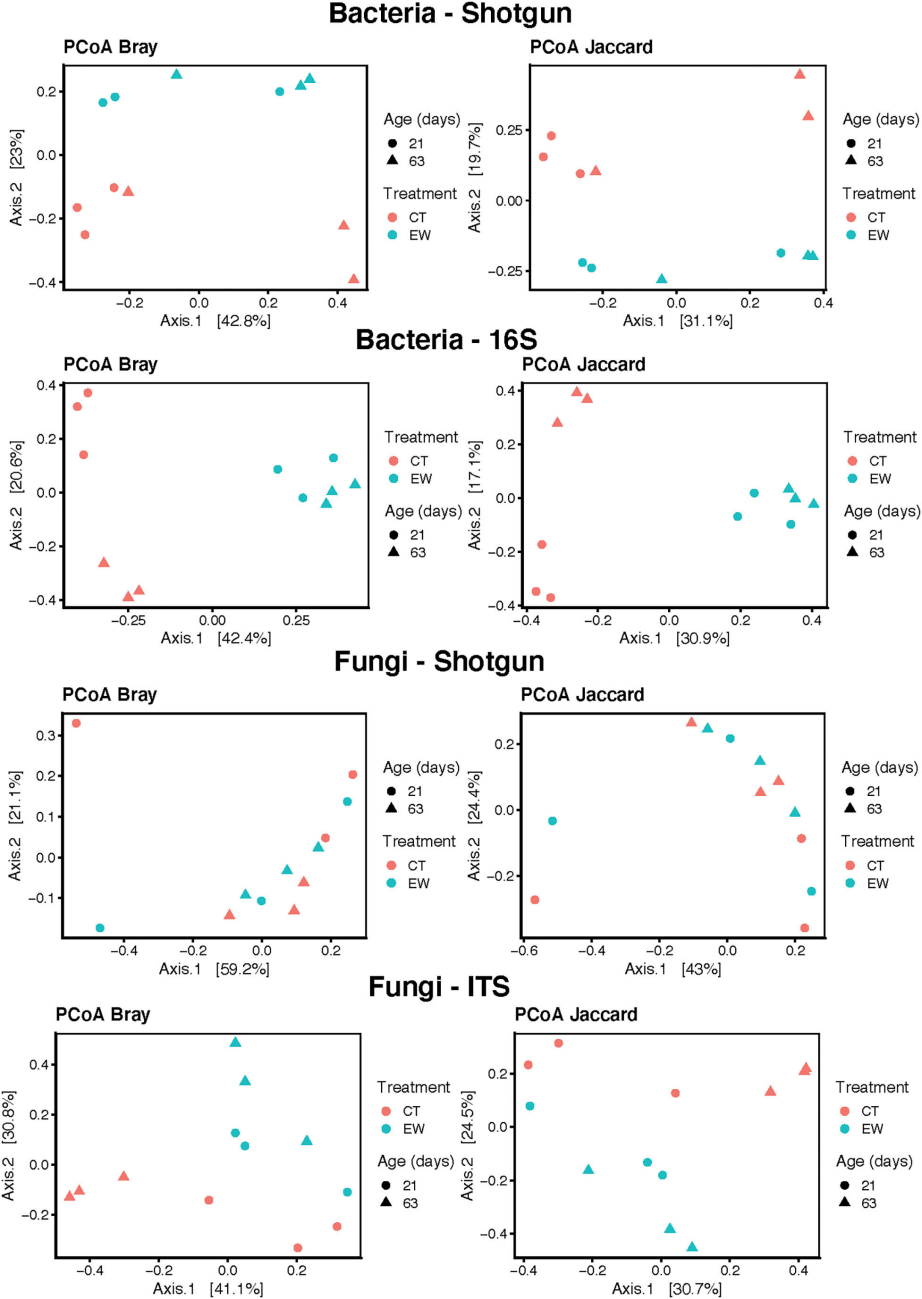
Bacterial PCoA plots of beta-diversity estimates (Bray-Curtis and Jaccard indices) for treatment (control, CT; earthworm, EW) and age (21 and 63 days) groups for shotgun metagenomic and 16S metataxonomic strategies.

Bacterial beta-diversity was estimated across treatments and ages using the Bray-Curtis and Jaccard distances and PCoA (Figure 3). Both indices clearly separated control (CT) and earthworm (EW) groups in both metagenomic and 16S rRNA sequencing strategies. These differences observed on the PCoA plots for treatment and age were then confirmed by our adonis analyses (0.05 < *P* < 0.0001) in both HTS approaches (Table 2). Moreover, the 16S rRNA PCoAs also separated well age groups in the CT samples compared to the shotgun approach, but none of the beta-diversity analyses of age groups were significant in our adonis analyses (Table 2).

**Figure 4:**
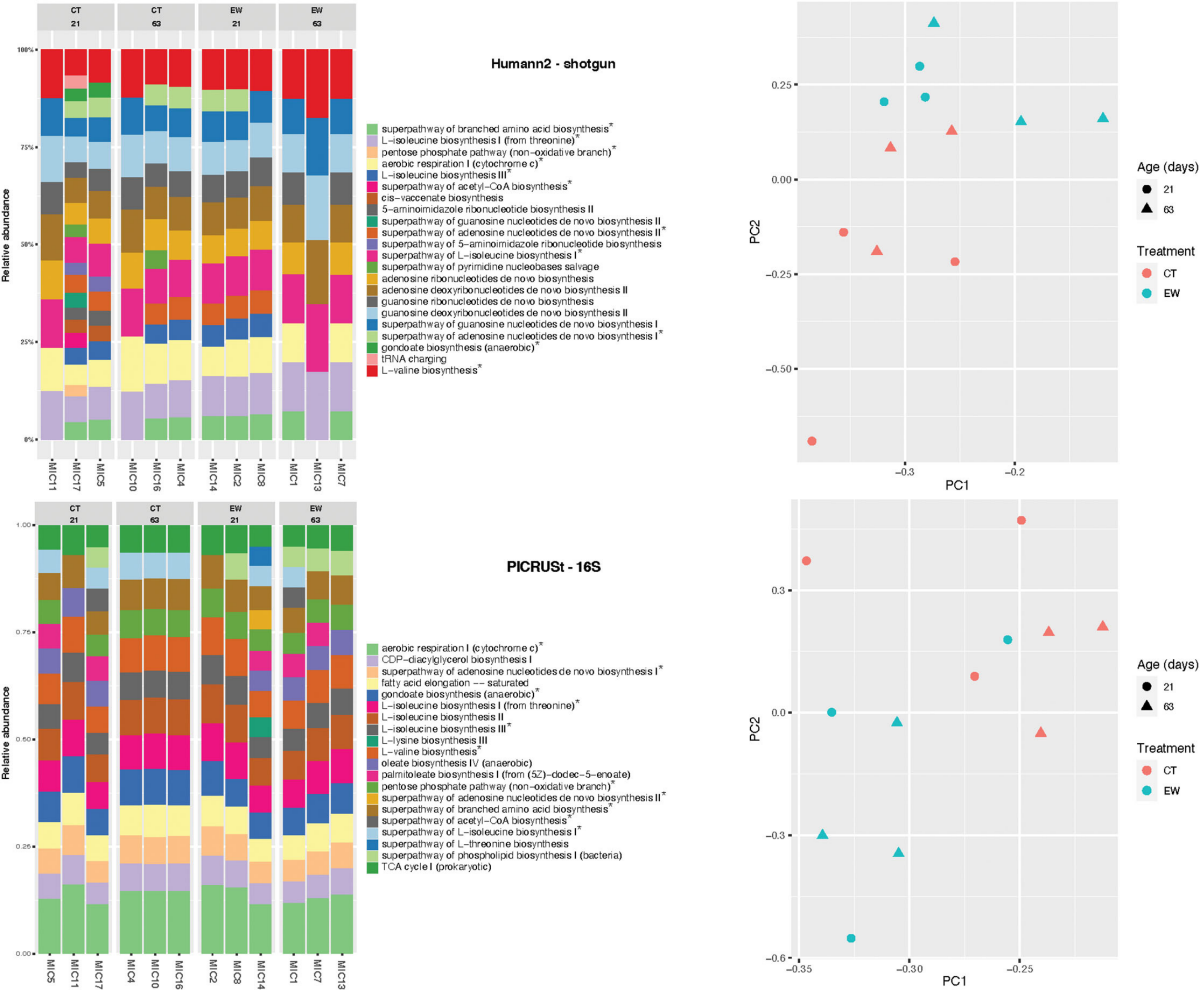
Bacterial functional profiles inferred by Humann2 using shotgun reads and predicted by PICRUSt2 using 16S amplicons in 12 microcosms (MIC). KEGG pathways found in both analyses are marked with an asterisk. PCA of pathways are also shown for both analyses.

Bacterial functional diversity was inferred and predicted using shotgun reads in Humann2 and 16S rRNA amplicons in PICRUSt2, respectively. The 23 and 20 most abundant KEGG metabolic pathways found by each approach are depicted in Figure 4. A total of 11 pathways were found by both approaches. The concordance between functional profiles was higher than between taxonomic profiles for the same bacterial samples. Nonetheless, community differences (beta-diversity) at the functional level in both approaches were sufficient to separate samples by treatment and age (Figure 4).

### Taxonomic Diversity of Mycobiomes

**Figure 5:**
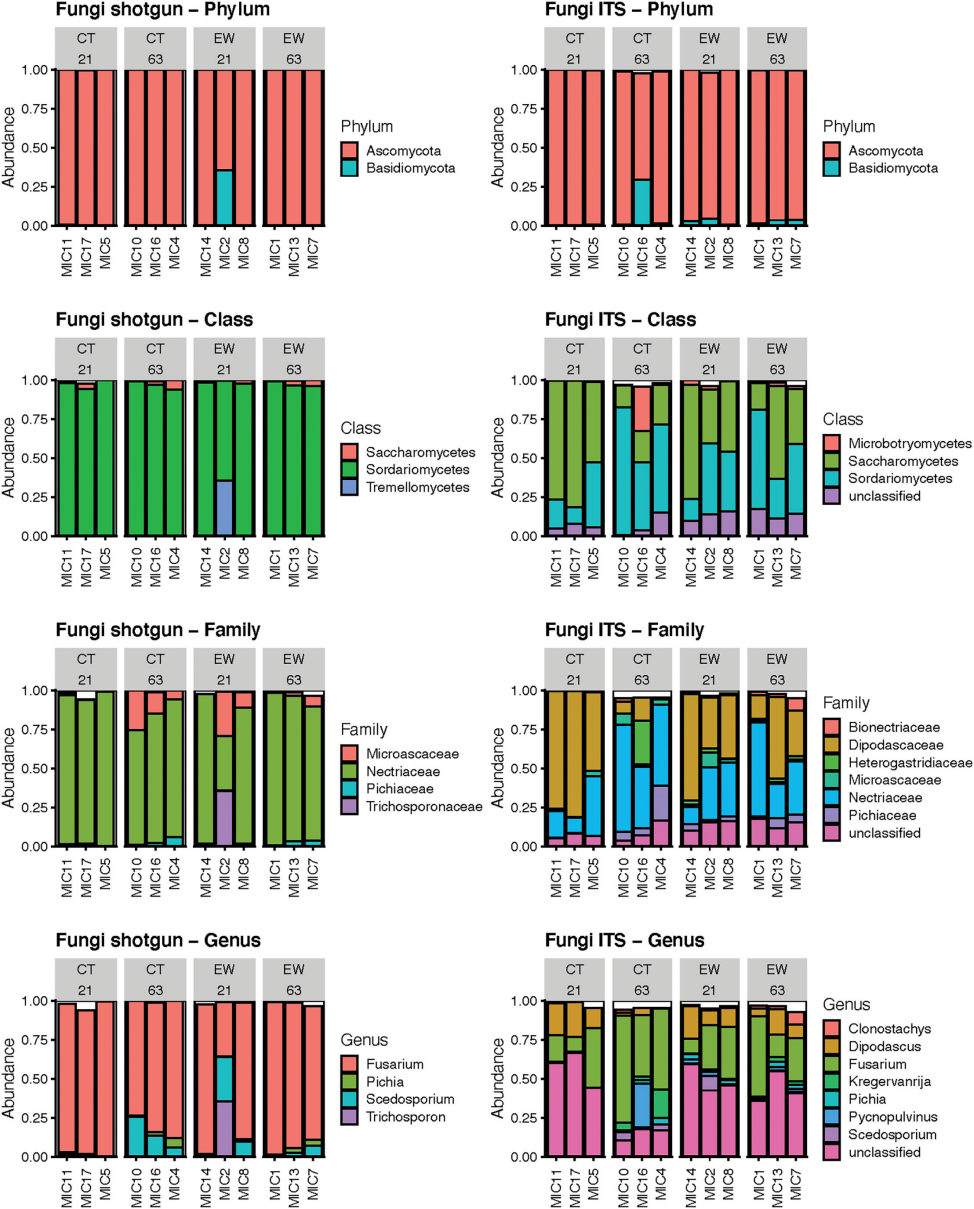
Bar plots of the mean relative abundance of the predominant fungi by phylum, class, family and genus using shotgun reads and ITS ASVs in 12 microcosms (MIC). The white section of the bar plots represents the less abundant taxa compiled.

The metagenomic approach detected 3 phyla, 9 classes, 18 families and 24 fungal genera; while the ITS amplicon approach detected 4 phyla, 11 classes, 32 families and 48 fungal genera. As before, we compared the rankings of mean relative abundances across those taxonomic categories between both HTS approaches (Figure 5; Supplementary Tables 4, 5). We observed less variation in rankings between HTS approaches for fungi than for bacteria, although the metagenomic strategy clearly showed less taxonomic variation than the metataxonomic approach. Both HTS methodologies depicted Ascomycota and Basidiomycota as the first and second most abundant fungal phyla. Sodariomycetes and Saccharomycetes ranked in the top two classes, while Tremellomycetes or Microbotryomycetes occupied the 3rd ranking (excluding the unclassified ITS ASVs). At the family level, Nectriaceae dominated in both HTS analysis, although in the ITS approach Dipodascaceae was similarly abundant and unclassified ASVs ranked 3rd. A similar pattern was observed at the genus level, where *Fusarium* occupied the 1st ranking among the classified sequences, but unclassified ASV showed higher abundance. As in the bacterial analyses, our ITS results also highlight the higher taxonomic resolution of the metagenomic approach for classifying fungal reads. Linear regression analyses of the mean relative abundance of the top two fungal phyla across treatment and age groups showed non-significant differences for both HTS approaches (Table 1).

**Figure 6:**
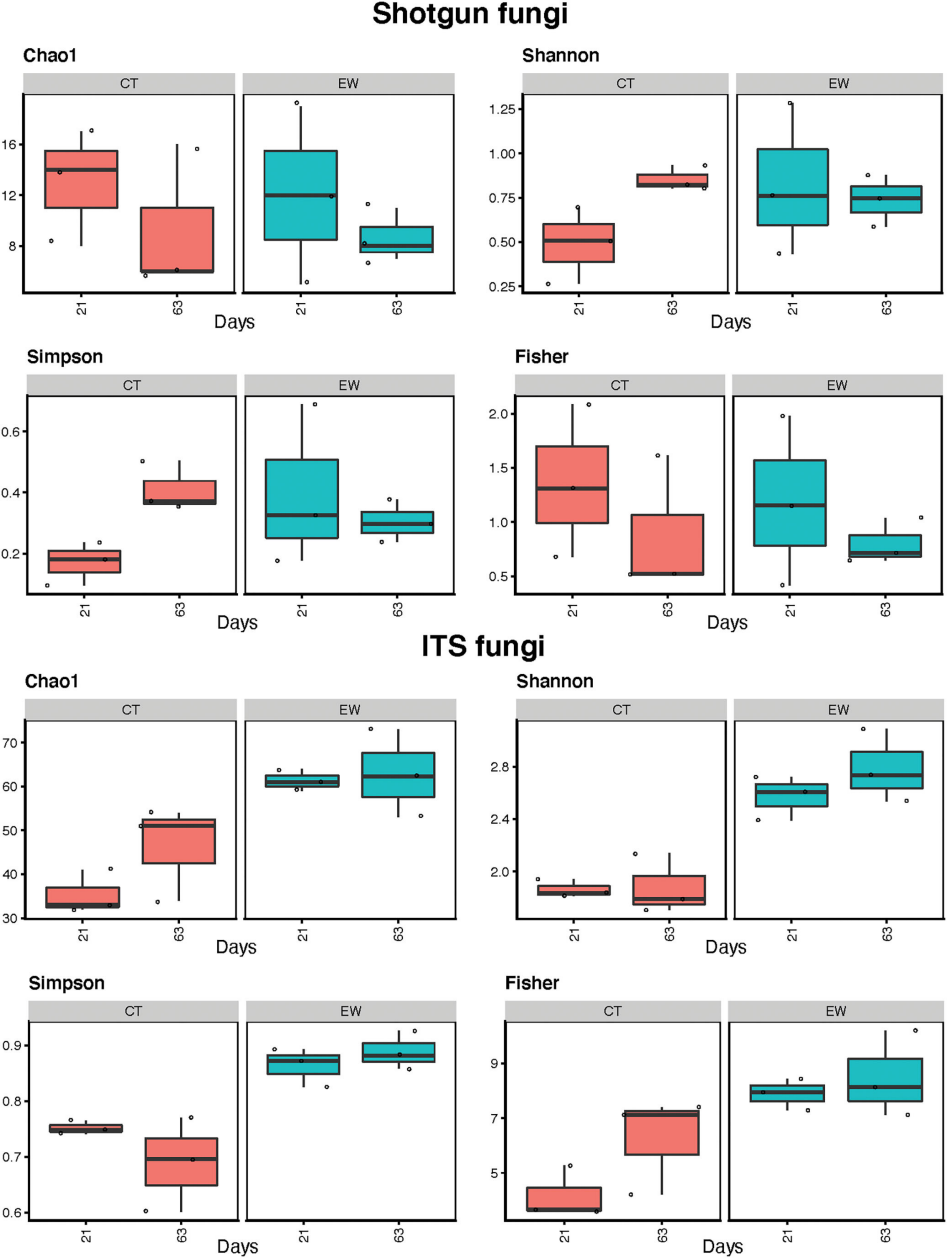
Fungal alpha-diversity estimates (Chao1, Shannon, Simpson, and Fisher indices) for treatment (control, CT; earthworm, EW) and age (21 and 63 days) groups for shotgun metagenomic and ITS metataxonomic strategies.

Fungal alpha-diversity estimates (Chao1, Shannon, Simpson, and Fisher) for treatment (CT and EW) and age (21 and 63 days) are depicted in Figure 6 and Table 2. Metagenomic data showed lower diversity estimates than ITS amplicon data for all groups. As for bacteria, diversity trends between metataxonomic and metagenomic approaches varied across indices and groups, with some even showing opposite trends (e.g., Chao1 CT) and with CT (no earthworms) showing the larger discrepancies. Overall fungal alpha-diversity increased or decreased over time in the CT groups, while it varied much less in the EW groups for all indices and HTS strategies. Moreover, in the metataxonomic approach, samples including earthworms showed higher levels of fungal diversity than controls (no earthworms) for all indices, but this trend was not observed in the metagenomic approach. Consequently, treatment was also an important (and significant) factor in all ITS comparisons of diversity, but it was not in any of the metagenomic comparisons. Age, however, varied significantly for two of the diversity indices in the metagenomic CT group (Table 2).

Fungal beta-diversity was estimated using the same indices (Bray-Curtis and Jaccard distances) and multidimensional scaling as its bacterial counterpart. Both PCoA plots clearly separated all samples of ITS ampicons by treatment and age (Figure 3). Metagenomic samples from both groups, however, were not well separated in the PCoA plots (Figure 3). These results were confirmed by our adonis analyses, which showed significant differences in the ITS approach for treatment and age, but not in the shotgun metagenomic approach (Table 2).

## Discussion

We compared two specific HTS approaches at estimating microbial taxonomy and function in compost (no earthworms) and vermicompost of white grape marc. We also used these data and analyses to better understand the dynamics of bacterial and fungal communities during vermicomposting.

### Taxonomic and Functional Diversity in Metagenomics and Metataxonomics

We sequenced the same 12 samples to compare the results of shotgun versus targeted amplicon sequencing. Since our samples represent natural populations, the real composition of their microbial communities is unknown; therefore, our experimental design, while allowing us to draw conclusions on HTS sensitivity, does not allow us to assess the specificity of each HTS method. This issue, however, goes beyond the scope of our study and has been already addressed by us and others using simulated NGS data and mock microbiotas (McIntyre et al., 2017; Sczyrba et al., 2017; Nasko et al., 2018; Heeger et al., 2019; Ye et al., 2019; Zielezinski et al., 2019; Marcelino et al., 2020; Miossec et al., 2020).

The 16S rRNA and ITS amplicon approaches have been the most commonly employed methods to analyze bacteriotas and mycobiotas, respectively. They have several important advantages compared to whole metagenome sequencing (WMS): (1) reduced cost, (2) low computational power and analytical speed, (3) extensive and curated reference databases (DeSantis et al., 2006; Quast et al., 2013; Nilsson et al., 2019b), and (4) well-established bioinformatic analytical pipelines (Schloss et al., 2009; Callahan et al., 2016; Bolyen et al., 2019). Shotgun sequencing of microbiotas, however, can provide a picture of the whole microbiota (and its host) and deeper resolution at lower taxonomic levels (Jovel et al., 2016). Our analytical results seem to confirm this last statement for the bacteriotas, since shotgun HTS identified 1.75 times more bacterial genera than 16S rRNA HTS. For fungal communities, however, ITS HTS identified two times more fungal genera than shotgun HTS. Our relatively low sequencing depth of the fungal kingdom (13–170 thousand reads/sample) and the larger size of their genomes (typically several orders of magnitude larger compared to bacterial genomes) may have jeopardized our ability to detect more fungal taxa in the metagenomic approach. However, a rarefaction analysis of the fungal shotgun data showed adequate sample sizes for all the samples (Supplementary Figure 1), indicating that we are recovering most of the microbial diversity. Other factors then, may have also contributed to exacerbate this discrepancy, including misclassification by the ITS approach and size differences in HTS reference databases. In fact, ITS primer biases and the uneven lengths of ITS fragments are known to promote preferential amplification of shorter sequences, leading to a biased quantification of taxon relative abundance and of alpha-diversity (Bellemain et al., 2010; De Filippis et al., 2017). A well-known limitation of the metagenomic approach, particularly when applied to the study of microbiotas of non-model systems, is the relatively low availability of fungal genome references. At the time our analyses were performed, there were 402 fungal genome references available in the RefSeq database (O'Leary et al., 2016) and ~1 million ITS references in the UNITE database (Nilsson et al., 2019b). The number of genomes is steadily increasing but is still far from being sufficiently comprehensive to capture a substantial portion of the fungal diversity present in most ecosystems (Lewin et al., 2018). While the use of ITS amplicon data has its own limitations and methodological issues, it could be expected to yield a more comprehensive catalog of taxa present in our samples due to the more established database resource currently available.

Microbial composition and abundance estimated by both HTS approaches agreed reasonably well at the phylum level, but larger discrepancies were observed at lower taxonomic levels (class to genera). This may be due to the higher resolution of the metagenomics approach (Hong et al., 2014; Lu and Salzberg, 2020) or potential primer bias issues with the metataxonomic approach (Klindworth et al., 2013; Eloe-Fadrosh et al., 2016; Laursen et al., 2017). Nonetheless, at all taxonomic levels the most abundant microbial taxon in the metagenomic approach was also the most abundant microbial taxon in the 16S rRNA and ITS metataxonomic approaches. It has been shown that taxonomic classification performed with metagenomic and metataxonomic approaches will be to some extent divergent, as the resolution of the sequences used for taxonomic assignments is distinct and variable depending on the genome region captured in shotgun surveys, the variable region of the targeted amplicon gene used, and the microbial composition the community under analysis (Jovel et al., 2016). Previous studies have shown a good concordance between both approaches for simpler bacterial communities, but higher discrepancies are expected in more complex microbiotas like ours.

Alpha diversity metrics summarize the structure of an ecological community with respect to its richness (number of taxonomic groups), evenness (distribution of abundances of the groups), or preferably both. Here we estimated four alpha-diversity indices for comparison purposes. Only the Chao1 richness index showed higher estimates for the shotgun approach than for the 16S rRNA amplicon approach, while the other three indices, which account for richness and evenness, showed similar or higher diversity for the latter method. Similarly, the ITS approach showed higher diversity for all indices than the shotgun approach. Multiple studies have shown that WMS yields higher levels of within-sample diversity than amplicon methods. But, a recent thorough comparative analysis of amplicon and metagenomic sequencing methods using 10 different animal hosts revealed that measures of alpha diversity can vary drastically (and significantly) between shotgun and 16S rRNA HST, with the former sometimes showing similar or lower richness and Shannon diversity than the latter; the study has also shown that such patterns seem to be mostly host-specific (Rausch et al., 2019). Similar comparative studies are still lacking for ITS vs. WMS, but one could expect comparable results to those seen for 16S rRNA vs. HTS by Rausch et al. (2019).

Alpha-diversity trends in HTS approaches also varied greatly between groups for the two compared factors (treatment and age), with some showing opposite (and sometimes significant) results. This is obviously of concern because one would reach different conclusions about the dynamics of microbial communities depending on the HTS approach used. Since the true microbial composition of our natural samples is unknown, we cannot determine what HTS approach better represents the true microbial dynamics. But as indicated above, since both approaches may target different layers of the microbial community, combining results from both metataxonomic and metagenomic approaches may actually provide a complementary and more comprehensive view of the microbiota under study.

Indices of microbial structure (beta-diversity) estimated by the amplicon approaches separated all samples by treatment and age, while the shotgun approach only segregated the bacteria and clustered most of the fungal samples. The fungal PCoAs plots (Figure 3) improved if the two most divergent samples (MIC2 and MIC17) were excluded, but still no clear pattern was discerned and the adonis analysis was not significant (data not shown). We suspect that the relatively low number of available fungal genome references in the RefSeq database may have limited our ability to discern patterns of microbial structure in the fungal shotgun data. Discrepancies in beta-diversity between metagenomic and 16S rRNA metataxonomic approaches for the same two distances used here have also been reported in the comparative study of Rausch et al. (2019), but no other study has so far applied both approaches to assess fungal structure in vermicompost microbiotas.

The metabolic potential of the microbiome can be predicted or cataloged from 16S rRNA and shotgun metagenomics libraries, respectively. Amplicon approaches like 16S rRNA rely on the correlation between phylogenetic trees and clusters of genes shared between taxa (Langille et al., 2013). Shotgun metagenomics, on the other hand, provides a direct assessment of the functional attributes of the microbiome (Knight et al., 2012). In our analyses, PICRUSt2-imputed functional repertoires overlapped ~50% with shotgun profiles. Previous studies have shown larger discrepancies between both approaches (Jovel et al., 2016; Rausch et al., 2019), while others have shown a good concordance (Langille et al., 2013). Variation in imputation success is largely dependent on the composition of the particular host community and the 16S rRNA region used, which suggests that PICRUSt2 profiles should be interpreted with caution (Rausch et al., 2019). Shotgun metagenomics then offers a more reliable assessment, although its accuracy depends on sequencing depth (Knight et al., 2012). Hence, ideally, both HTS approaches should be complemented with actual metabolic profiles generated *via* metatranscriptomics and confirmed by proteomics (Franzosa et al., 2015).

### Microbial Dynamics During Vermicomposting of White Grape Marc

Our HTS analyses have revealed differences in composition, structure, and diversity (taxonomic and functional) over time in the bacteriotas and mycobiotas of compost microcosms inoculated with earthworms and controls (no earthworms). Previous studies of the bacterial microbiome during vermicomposting of different plant materials such as grape marc (Gomez Brandon et al., 2019; Kolbe et al., 2019) and the leguminous shrub Scotch broom (Dominguez et al., 2019) also showed significant changes in community composition, structure, and metabolic potential. They also demonstrated that bacterial communities change quickly and dramatically during the first weeks of vermicomposting. Those studies, however, did not include free-earthworm microcosms followed over time, as we did here; hence they could not untangle the compound effect of the earthworms from that of the microbes in the successional changes that take place during decomposition. Our results here show and confirm that although microorganisms (bacteria and fungi) are the main agents of biochemical decomposition of dead organic matter (grape marc), earthworms are also key drivers in plant vermicomposting, accelerating decomposition and drastically modifying physical and microbiological properties of the substrate (Domínguez et al., 2010). Dominguez et al. (2021) have shown in the vermicomposting of sewage sludge, that the earthworm gut can eliminate up to 96 and 91% of the ingested bacterial and fungal taxa, respectively. Gut transit is also responsible for the drastic microbial changes we observed here in the first few weeks of grape marc vermicomposting. Both metagenomic and metataxonomic analyses were able to reveal those changes, but metagenomics is more informative, has the potential for a finer degree of resolution and may help us to unravel worm-microbe interactions.

Our comparative study of the same 12 natural samples (microcosms) pinpoints similarities and differences between the specific amplicon and shotgun HTS approaches and analytical pipelines used for the characterization of bacteriotas and mycobiotas. Other workflows may lead to different results and inferences than those presented here. Our study also provides a complementary and comprehensive view of the diversity and temporal dynamics of microbial communities during vermicomposting of white grape marc. We hope our results highlight the strengths and weaknesses of each approach and stimulate further microbiome research on vermicomposting.

## Data Availability Statement

The datasets presented in this study can be found in online repositories. The names of the repository/repositories and accession number(s) can be found below: NCBI BioProject—PRJNA777435.

## Author Contributions

MP-L and JD designed and performed research. MP-L, AK and IR-T analyzed data. MP-L wrote the first draft of the manuscript. KC, MP-L, and JD tasted the wine. All authors read, revised and approved the final draft of the manuscript.

## Funding

This study was co-funded by EU via European Regional Development Fund (ERDF) and by national funds via FCT via the project PTDC/ASP-PES/27953/2017 - POCI-01-0145-FEDER-027953. It was also supported by the Spanish Ministerio de Economía y Competitividad (AGL2017-86813-R) and the UE program H2020 (LABPLAS_101003954). MP-L was supported by FCT under the Programa Operacional Potencial Humano - Quadro de Referência Estratégico Nacional funds from the European Social Fund and Portuguese Ministério da Educação e Ciência IF/00764/2013. EC-N was funded by ANIDFONDECYT Regular 1200834 and by ANID-PIA-Anillo INACH ACT192057.

## Conflict of Interest

The authors declare that the research was conducted in the absence of any commercial or financial relationships that could be construed as a potential conflict of interest.

## Publisher's Note

All claims expressed in this article are solely those of the authors and do not necessarily represent those of their affiliated organizations, or those of the publisher, the editors and the reviewers. Any product that may be evaluated in this article, or claim that may be made by its manufacturer, is not guaranteed or endorsed by the publisher.

## Supplementary Material

Supplementary Figure 1 Rarefaction curves indicating the number of amplicon sequence variants (ASVs) identified in 16S and ITS datasets and species identified in shotgun datasets in 12 microcosms (MIC).

Supplementary Table 1 Generated raw and filtered reads (after QC) for shotgun and amplicon high throughput sequencing.

Supplementary Table 2 Mean relative abundance of bacterial shotgun reads by phylum, class, family and genus. MIC, microcosm.

Supplementary Table 3 Mean relative abundance of bacterial 16S ASVs by phylum, class, family and genus. MIC, microcosm.

Supplementary Table 4 Mean relative abundance of fungal shotgun reads by phylum, class, family and genus. MIC, microcosm.

Supplementary Table 5 Mean relative abundance of fungal ITS ASVs by phylum, class, family and genus. MIC, microcosm.

## Abbreviations

ASV: Amplicon sequence variant
CT: Control
DNA: Deoxyribonucleic acid
EW: Earthworm
HTS: High-throughput sequencing
ITS: Internal transcribed spacer
KEGG: Kyoto encyclopedia of genes and genomes
KO: KEGG ortholog
LM: Linear models
MIC: Microcosm
MG: metagenomics
NGS: Next-generation sequencing
OTU: Operational taxonomic unit
PCA: Principal component analysis
PCoA: Principal coordinate analysis
PCR: Polymerase chain reaction
rRNA: Ribosomal ribonucleic acid
WMS: Whole metagenome sequencing.
