# Supplementary figures and images for: Comparative Analysis of Metagenomics and Metataxonomics for the Characterization of Vermicompost Microbiomes
Source: Front Microbiol. 2022 May 10;13:854423. doi: 10.3389/fmicb.2022.854423 (PMC9127802; doi:10.3389/fmicb.2022.854423)

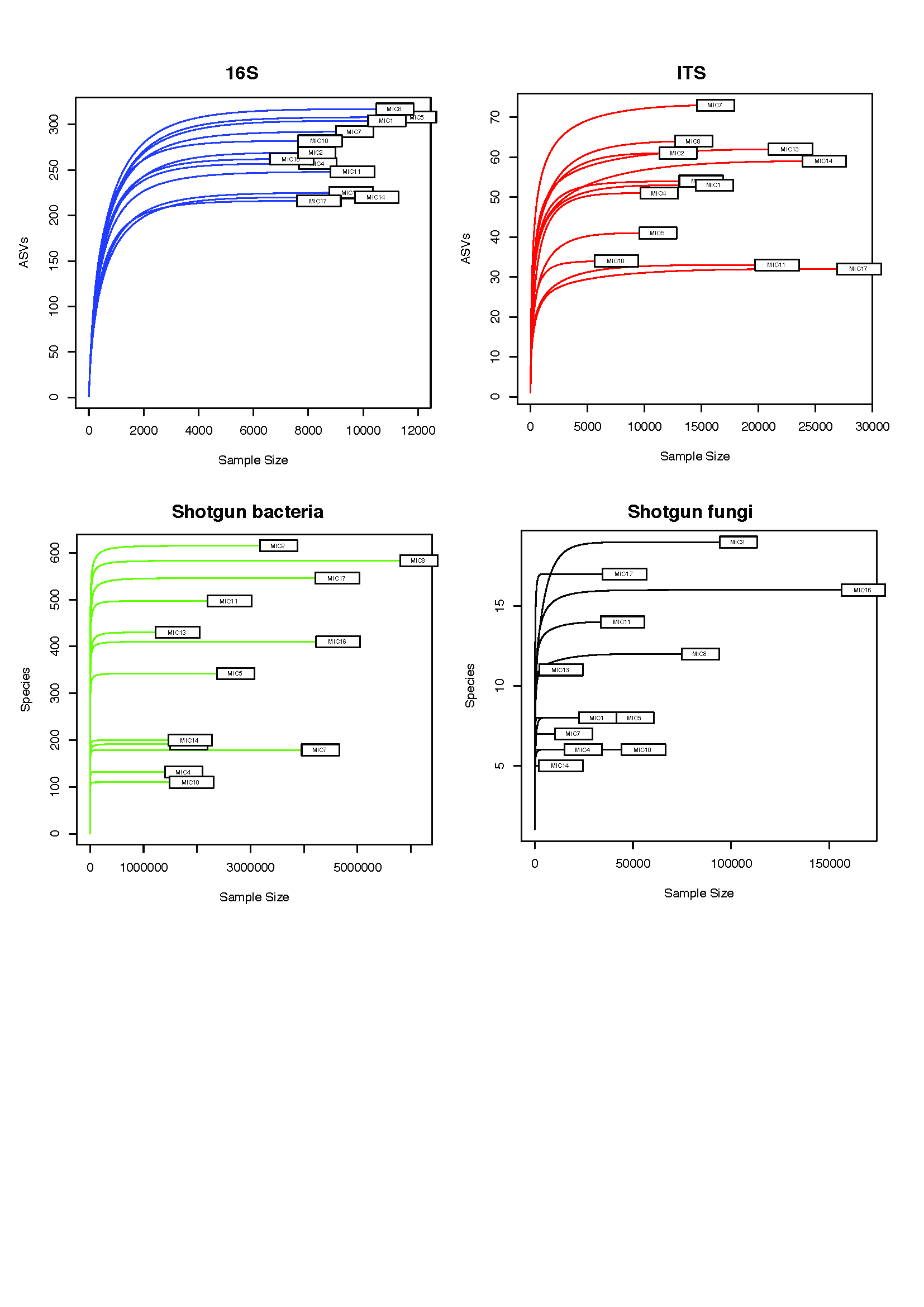

Supplement: Supplementary Figure 1 — Rarefaction curves indicating the number of amplicon sequence variants (ASVs) identified in 16S and ITS datasets and species identified in shotgun datasets in 12 microcosms (MIC). [file Image_1.tiff]
